# What contributes to an effective mannose recognition domain?

**DOI:** 10.3762/bjoc.13.255

**Published:** 2017-12-04

**Authors:** Christoph P Sager, Deniz Eriş, Martin Smieško, Rachel Hevey, Beat Ernst

**Affiliations:** 1 Department of Pharmaceutical Sciences, University of Basel, Klingelbergstrasse 50, CH-4056 Basel, Switzerland

**Keywords:** carbohydrate–lectin interactions, desolvation penalty, dielectric constant, multivalency, pre-organization

## Abstract

In general, carbohydrate–lectin interactions are characterized by high specificity but also low affinity. The main reason for the low affinities are desolvation costs, due to the numerous hydroxy groups present on the ligand, together with the typically polar surface of the binding sites. Nonetheless, nature has evolved strategies to overcome this hurdle, most prominently in relation to carbohydrate–lectin interactions of the innate immune system but also in bacterial adhesion, a process key for the bacterium’s survival. In an effort to better understand the particular characteristics, which contribute to a successful carbohydrate recognition domain, the mannose-binding sites of six C-type lectins and of three bacterial adhesins were analyzed. One important finding is that the high enthalpic penalties caused by desolvation can only be compensated for by the number and quality of hydrogen bonds formed by each of the polar hydroxy groups engaged in the binding process. In addition, since mammalian mannose-binding sites are in general flat and solvent exposed, the half-lives of carbohydrate–lectin complexes are rather short since water molecules can easily access and displace the ligand from the binding site. In contrast, the bacterial lectin FimH benefits from a deep mannose-binding site, leading to a substantial improvement in the off-rate. Together with both a catch-bond mechanism (i.e., improvement of affinity under shear stress) and multivalency, two methods commonly utilized by pathogens, the affinity of the carbohydrate–FimH interaction can be further improved. Including those just described, the various approaches explored by nature to optimize selectivity and affinity of carbohydrate–lectin interactions offer interesting therapeutic perspectives for the development of carbohydrate-based drugs.

## Review

### Recognition of carbohydrate ligands

For the recognition of carbohydrate ligands, nature has explored binding sites of different shapes and properties. The large family of C-type lectins (CLECs) exhibits carbohydrate-recognition domains (CRDs) which incorporate a calcium ion [1–4]. CLECs are involved in a wide range of biological processes, such as pathogen recognition and intercellular adhesion [5–7]. A large number of CLEC structures, including animal, plant and bacterial lectins, are available in the Protein Data Bank [8]. A second large family of lectins, the bacterial adhesins, play an important role in the initial interaction of the bacterium with host tissue [9–10]. This primary contact is a prerequisite for the infection of host cells and subsequent biofilm formation, and grants the bacteria a significant advantage by resisting clearance and killing by immune factors, bacteriolytic enzymes, or antibiotics.

In this review, with focus on lectins relevant for drug discovery and development, the mannose-binding sites of six CLECs and three bacterial lectins are analyzed and compared with one another to answer the question: What makes for a successful mannose recognition domain? In general, lectins are characterized by high ligand specificity, whereas the affinity for their carbohydrate ligands is comparatively low. A prominent example is sialyl Lewis^x^ (sLe^x^), a tetrasaccharide typically O-linked to cell surfaces and known to play a vital role in cell-to-cell recognition processes [11]. Although highly specific, its interaction with E-selectin exhibits a dissociation constant (*K*_D_) of only 800 μM [12]. To address this obstacle of low affinity, nature applies the principal of multivalency by providing several binding sites to the carbohydrate ligand and/or a multivalent display of the ligand [13–15]. This accumulation of individual binding events increases the overall binding strength either by avidity or local concentration effects [16–17]. However, other approaches, such as the reduction of desolvation costs or ligand and binding site pre-organization, are more difficult to assess and accordingly have been highlighted in this review.

Mannose-binding CLECs are involved in various pathways of the human innate immune response, including the blood dendritic cell antigen 2 (BDCA-2, also known as CD303) [18], langerin (CD207) [19–20], pulmonary surfactant-associated protein D (SP-D) [21], dendritic cell-specific ICAM-3-grabbing non-integrins 1 and 2 (DC-SIGN, also known as CD209; and DC-SIGNR, also known as CD299) [22–23], and mannose-binding protein (MBP) [24]. These CLECs exert their function through different mechanisms, for instance by pathogen internalization as in the case of BDCA-2 and langerin, by pathogen opsonization as mediated by SP-D and MBP, or by T-cell interactions as mediated by DC-SIGN and DC-SIGNR [25–26].

In contrast, pathogens have developed numerous adhesins that mediate their interaction with glycosides on mammalian cell surfaces. After this initial contact, they can infect host cells and form biofilms, both of which are key factors for their survival [9,27–28]. Examples of such opportunistic bacterial species binding to mannosides on host cells include *Pseudomonas aeruginosa* with its membrane lectin LecB [29–30] and *Burkholderia cenocepacia* with its characteristic *B. cenocepacia* lectin A (BC2L-A) [31–32], both playing an important role in the social life of bacterial cells. A further example is the bacterial adhesin FimH, which plays a crucial role in urinary tract infections (UTIs). FimH enables uropathogenic *Escherichia coli* (UPEC) to adhere to urothelial host cells [33–34], which represents the first and most critical step in UTI, triggering a cascade of pathogenic processes ultimately leading to infection. The ligand on urothelial cells binding to the N-terminal lectin domain of FimH is the highly mannosylated glycoprotein uroplakin 1a (UPIa) [35–36]. The binding pocket of FimH accommodates a single α-D-mannose (**1**) with an extended hydrogen-bond network [37–38]. Accordingly, any modifications on the hydroxy groups of the mannose virtually abolish binding affinity [37–39].

### Crystal structures of mannose–lectin complexes

**Figure 1:**
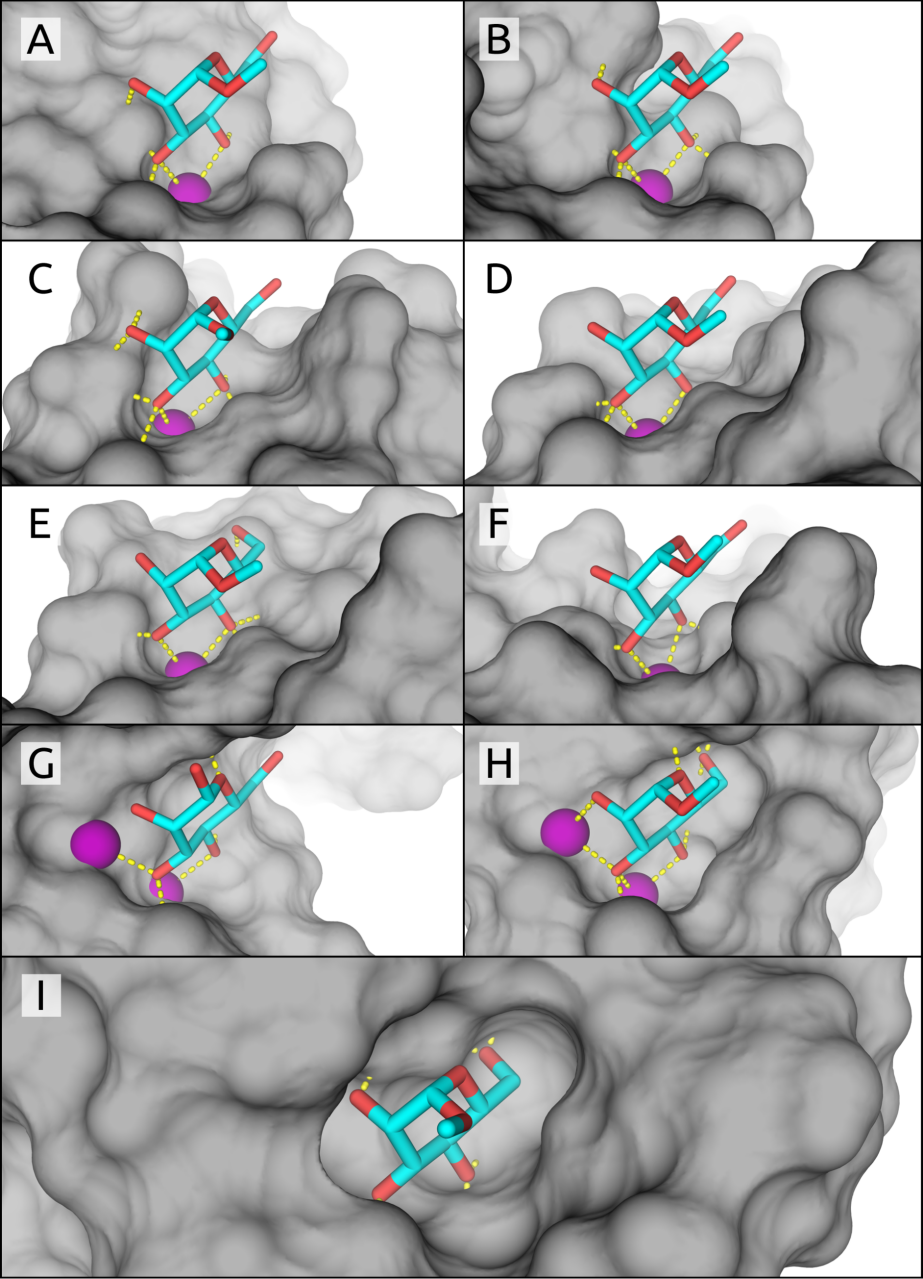
CRDs of the analyzed crystal structures, with mannose pyranosyl units similarly aligned in each structure. Mammalian lectins: (**A**) BDCA-2, (**B**) langerin, (**C**) SP-D, (**D**) DC-SIGNR, (**E**) DC-SIGN, (**F**) rat MBP; bacterial lectins: (**G**) LecB, (**H**) BC2L-A, and (**I**) FimH lectin domain.

The X-ray structures of six mannose-binding receptors in complex with either α-D-mannose (**1**) or methyl α-D-mannopyranoside (**2**) were analyzed (Figure 1 and Table 1, **A**–**C** and **G**–**I**). Since for DC-SIGNR (Figure 1, **D**) and DC-SIGN (Figure 1, **E**) neither complexes with **1** nor **2** were available, we instead modeled the monosaccharide–receptor interactions based on the available oligomannose crystal structures (PDB codes: 1K9J and 1SL4). In addition, because none of the available crystal structures of human MBP met our threshold of a resolution below 2 Å, we used a structure based on a homologous MBP lectin domain from *Rattus norvegicus* and accordingly compared the measured binding affinity of rat MBP (Figure 1, **F**). Finally, a special case is the bacterial adhesin FimH, which can adopt three different affinity states (see below). For our discussion we focus specifically on the high-affinity state of FimH present in the isolated lectin domain of FimH, called FimH_LD_ (Figure 1, **I**).

**Table 1:** Crystal structures of mannose-binding lectins, and their affinity for α-D-mannose (**1**) or methyl α-D-mannopyranoside (**2**).

lectin	target	affinity [µM]	ligand efficiency	PDB code	resolution [Å]	reference

**A**	BDCA-2	9.4 × 10^3 a^	0.22	4ZES	1.65	[40]
**B**	langerin	4.4 × 10^3 a^	0.25	4N37	2.00	[41]
**C**	SP-D	3.8 × 10^3 b^	0.28	3G81	1.80	[42]
**D**	DC-SIGNR	2.5 × 10^3 b^	0.30	1K9J^c^	1.90	[43,46]
**E**	DC-SIGN	2.3 × 10^3 b^	0.31	1SL4^c^	1.55	[22,43]
**F**	rat MBP	1.3 × 10^3 b^	0.34	1KWU	1.95	[44,47]
**G**	LecB	71^a^	0.45	1OUR	1.42	[29,45]
**H**	BC2L-A	2.8^a^	0.60	2VNV	1.70	[31]
**I**	FimH_LD_	1.2^a^	0.61	5JCQ	1.60	[48]

^a^Affinity of methyl α-D-mannoside (**2**); ^b^affinity of α-D-mannose (**1**); ^c^modified oligopyranomannose crystal structure.

Although the receptors **A**–**F** play important roles in human immune responses, they exhibit affinities only in the millimolar range (9.4–1.3 mM) for α-D-mannose (**1**) and methyl α-D-mannopyranoside (**2**) [40–44]. In contrast, the receptors **G** and **H** of bacterial origin show affinities in the micromolar range (71 and 2.8 µM, respectively) for methyl α-D-mannose (**2**) [31,45]. Despite the 71 µM affinity, LecB (**G**) preferably binds L-fucose (3 µM) and methyl α-L-fucoside (0.4 µM) [45]. The enhanced affinity for fucosides originates from the C5-methyl group, absent in both **1** and **2**, which can form a hydrophobic contact with Thr45 [45].

The analyzed CLECs **A**–**F** share a common binding motif, with a calcium ion coordinating to O–C3 and O–C4 of the mannose ligand [5,7]. In instances where the binding site hosts a second calcium ion (**G** and **H**), advantageous interactions between O–C2 and O–C3 can also occur. Additional contributions from H-bonds formed in the buried binding pockets further improve affinity. In contrast, the calcium-free, buried binding site of the bacterial lectin FimH (**I**) forms a complex network of eight hydrogen bonds with ligand **2**, one of them mediated by a conserved water [37].

### Various approaches to realize binding affinity

The immense variability of binding affinities among mannose-binding receptors is remarkable, albeit not surprising. While CRDs involved in the human immune system (Table 1, **A**–**F**) recognize a broader spectrum of binding partners (i.e., various pathogenic oligosaccharides), bacterial CRDs **G**–**I** strive for tight binding to host glycans to improve their chances of survival. To achieve these enhanced affinities, pathogens apply a variety of strategies such as binding sites with minimal solvent-exposed surface areas, increased number of ligand interactions, “shared” desolvation costs, and multivalency.

**Figure 2:**
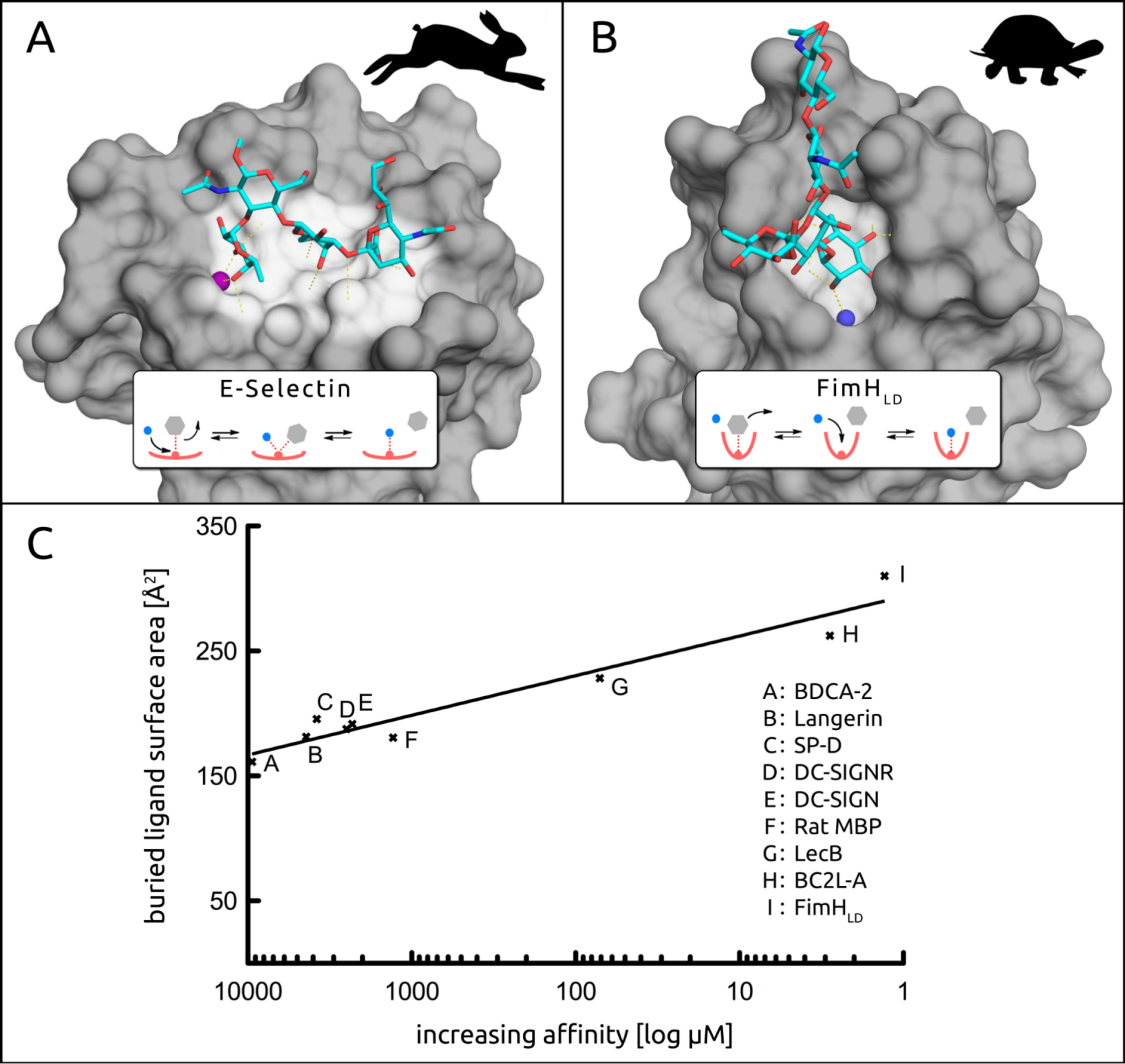
A) The solvent exposed binding site of E-selectin interacting with sLe^x^ (PDB: 1G1T) [53]. B) The buried binding site of FimH_LD_ in complex with a high-mannose epitope and a conserved water (blue sphere) (PDB: 2VCO) [36]. C) The buried ligand surface area of analyzed crystal structures correlates to affinity [μM] on a logarithmic scale (for references see Table 1). The rabbit and turtle metaphor was adapted from Schmidtke et al. [50].

**Degree of solvent exposure in the binding site** (Figure 2). Because of the electrostatic character of H-bonds, the dielectric constant ε becomes especially important in carbohydrate–lectin interactions. In buried cavities of the binding site, ε is lower (ε ≈ 5–10) compared to protein surfaces (ε ≈ 20) or bulk water (ε ≈ 80), making an H-bond thermodynamically up to 10-fold more valuable in buried cavities [49]. This at least partially explains the generally weak interactions of carbohydrates that bind on the solvent exposed surface of proteins, as compared to those of the majority of marketed drugs that most frequently bind to protein cavities. Additionally, buried and less solvent exposed ligands show slower exchange rates, characterized by a high-energy transition state. This can be explained by the stepwise dissociation and subsequent rehydration that are required for ligand displacement (inset, Figure 2B), due to the inherently shielded nature of the buried binding site. In contrast, solvent exposed H-bonds can be more easily substituted by surrounding water molecules in a concerted, bimolecular process (inset, Figure 2A), resulting in faster off-rates and therefore poor pharmacodynamics [50–51]. Similarly, water molecules in buried binding sites show residence times in the micro- to nanosecond range as opposed to surface water molecules which exhibit short residence times in the low picosecond range [52].

Whereas E-selectin in complex with sLe^x^ is an excellent example of a solvent exposed interaction [12,53], the interaction of FimH_LD_ with mannosides well illustrates the counter situation for a deep CRD [36] (Figure 2A and B, respectively). This difference in solvent exposure leads to considerably different residence times for their physiological ligands. Whereas sLe^x^ has a residence time of less than a second, the natural substrate of FimH_LD_ (**I**) displays a residence time of more than a minute, and for some synthetic FimH_LD_ antagonists even longer [48,54].

Among the analyzed CLECs **A**–**F** and bacterial lectins **G**–**I**, affinity increases with a decrease in solvent exposure of the binding site (Figure 2C). The buried ligand surface area, an alternative way of expressing solvent exposure of the binding site, is between 160–180 Å^2^ for **A**–**F**, 228 Å^2^ for **G**, 262 Å^2^ for **H**, and 310 Å^2^ for **I**. The decreased dielectric constant ε in the deep cavities of **H** and **I**, as well as the resulting occlusion of the ligand from surrounding water molecules, leads to a more stable hydrogen-bond network and thus to higher affinities. Furthermore, the binding site of **F** features the aromatic His189, that can engage in CH–π interactions, associated with contributions to the binding affinity in the range of 0–6.3 kJ/mol [55–56].

**Figure 3:**
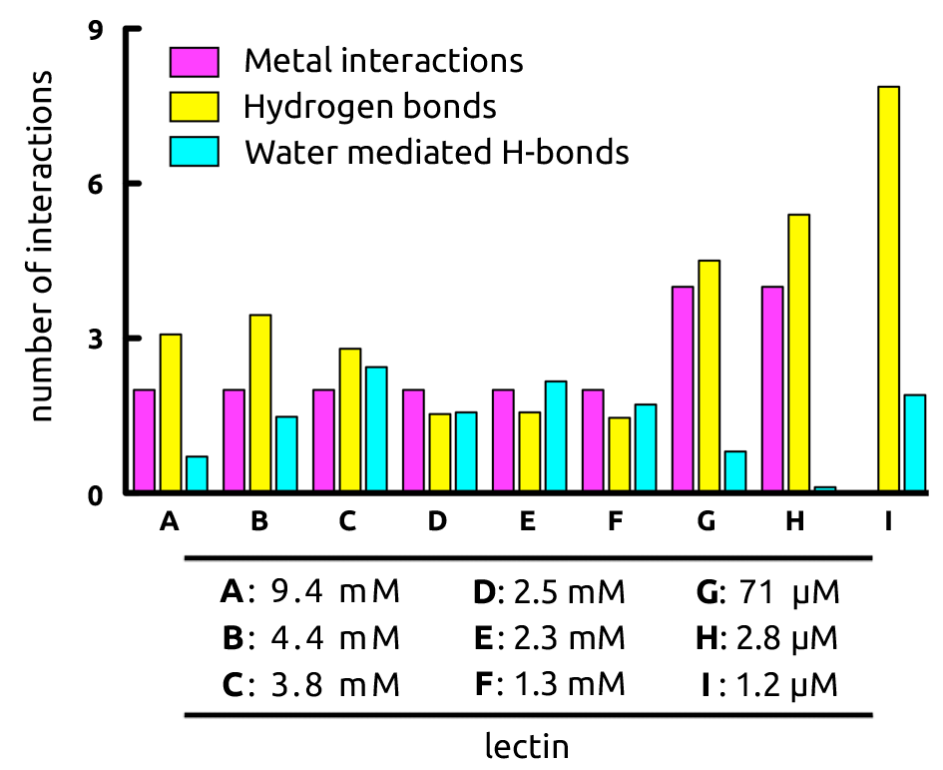
Dynamic mannose–receptor interactions (20 ns MD simulations), grouped according to the nature of the interaction. Metal interactions with Ca^2+^ are colored in purple, hydrogen bonds highlighted in yellow, and water-mediated interactions in cyan. (**A**) BDCA-2, (**B**) langerin, (**C**) SP-D, (**D**) DC-SIGNR, (**E**) DC-SIGN, (**F**) rat MBP, (**G**) LecB, (**H**) BC2L-A, (**I**) FimH_LD_.

**Analysis of the dynamics of mannose–lectin interactions** (Figure 3). In a next step, the stability of H-bond and metal interactions, as well as the influence of highly mobile vs conserved waters were analyzed. For the assessment of the dynamic behavior of the ligand complexes of the seven calcium-dependent lectins, 20 ns molecular dynamics (MD) simulations were performed [57]. The most prominent interactions of O–C3 and O–C4 of the mannose moiety with the calcium ion of CLECs **A**–**F** were stable throughout the entire simulation [5,7]. With the bacterial lectins LecB (**G**) and BC2L-A (**H**) each featuring two calcium ions the carbohydrate ligand forms up to four interactions: O–C2 and O–C4 provide one each, while O–C3 engages with both calcium ions.

During MD simulations, the number of ligand–protein hydrogen-bond interactions for lectins **A**–**F** varied from 1.5 to 3.5, and subsequently increased to 4.5 and 5.4 for LecB (**G**) and BC2L-A (**H**), respectively. Lastly, FimH (**I**) forms on average 7.9 hydrogen bonds with methyl α-D-mannopyranoside (**2**). For H-bonds that were only partially present during the MD simulation, non-integer numbers of hydrogen bonds arise.

The number of water-bridged H bonds between ligand and lectin varied greatly (Figure 3), from 0.1 to 2.4 for the buried binding site of BC2L-A (**H**) versus the solvent exposed binding site of SP-D (**C**), respectively. Interestingly, although the structurally similar bacterial CRDs of **G** and **H** differ by only one amino acid in the β8-β9-loop, a large impact on the number of water-mediated H-bonds was observed. Thus, Thr98 in the bacterial lectin **G** allows for the entry of a water molecule close to the first calcium ion, a process which is hindered by His112 in **H**, leading to a 25-fold difference in affinity. However, in the case of highly mobile water molecules, water-mediated H-bonds as observed in MD simulations destabilize the carbohydrate–lectin interaction, whereas a pre-constrained water molecule does not lead to an additional entropy penalty upon H-bonding to the ligand. As a result, the interaction benefits from an enthalpic gain without suffering from an entropic penalty [58]. Examples of such highly conserved water molecules are found in both, L-arabinose binding protein (ABP) [59] and FimH (**I**), where in the latter the water mediated H-bond originates solely from one stable water interacting with O–C2 (Figure 2B).

**Figure 4:**
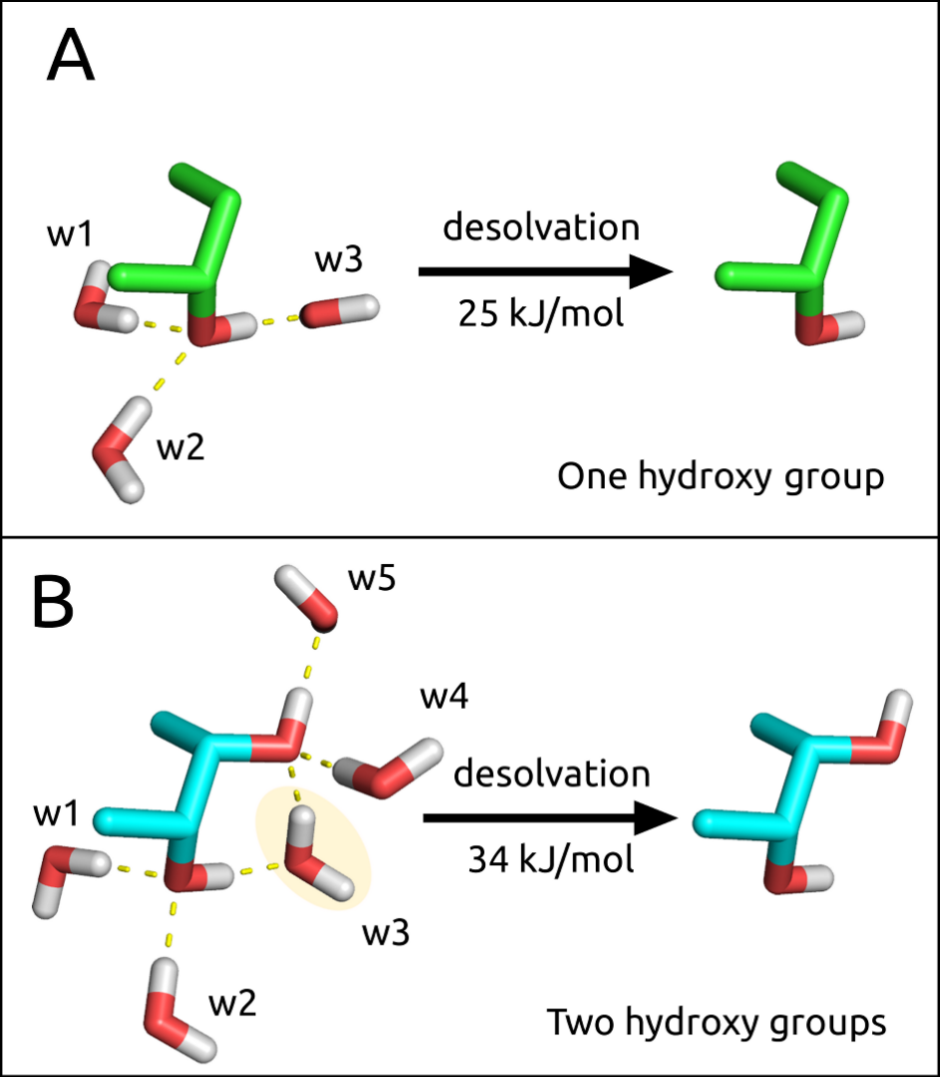
Desolvation of hydroxy groups. A) The desolvation cost of a single hydroxy group associated with three water molecules is Δ*G* = 25 kJ/mol, as calculated by Cabani et al. [61]. B) the desolvation cost of two adjacent hydroxy groups associated with five water molecules is Δ*G* = 34 kJ/mol, as opposed to 50 kJ/mol (equal to twice 25 kJ/mol). This can be explained by water w3, which is shared between the two hydroxy groups.

**The cost of desolvating hydroxy groups** (Figure 4). In general, when the low affinity issue regarding carbohydrate–lectin interactions is discussed, the costs of desolvation are often neglected. Because of the large number of hydroxy groups present in carbohydrate ligands, and the polar amino acid side chains of the lectin binding sites, desolvation generates an essential enthalpic penalty which can hardly be compensated for by the newly formed electrostatic interactions [60]. Cabani et al. calculated that the desolvation of an isolated hydroxy group causes an enthalpic penalty of Δ*H* = 35 kJ/mol, which is slightly reduced by a beneficial entropic term of Δ*S* = 10 kJ/mol due to the release of solvating water molecules into bulk [61]. As a result, the desolvation penalty for one hydroxy group amounts to Δ*G* = 25 kJ/mol (Figure 4A) and cannot be compensated for by a single hydroxy H-bond, which has been associated with a maximal energy gain of approximately Δ*G* = 18 kJ/mol [62–63]. However, for vicinal hydroxy groups as are present in carbohydrate ligands, the overall desolvation penalty is slightly reduced resulting in an overall desolvation cost of Δ*G* = 34 kJ/mol for both hydroxy groups (Figure 4B). Since carbohydrates in general exhibit a number of adjacent hydroxy groups, their desolvation penalties are difficult to assess but it is most likely that each additional hydroxy group would not contribute the maximum penalty associated with an isolated one.

**Figure 5:**
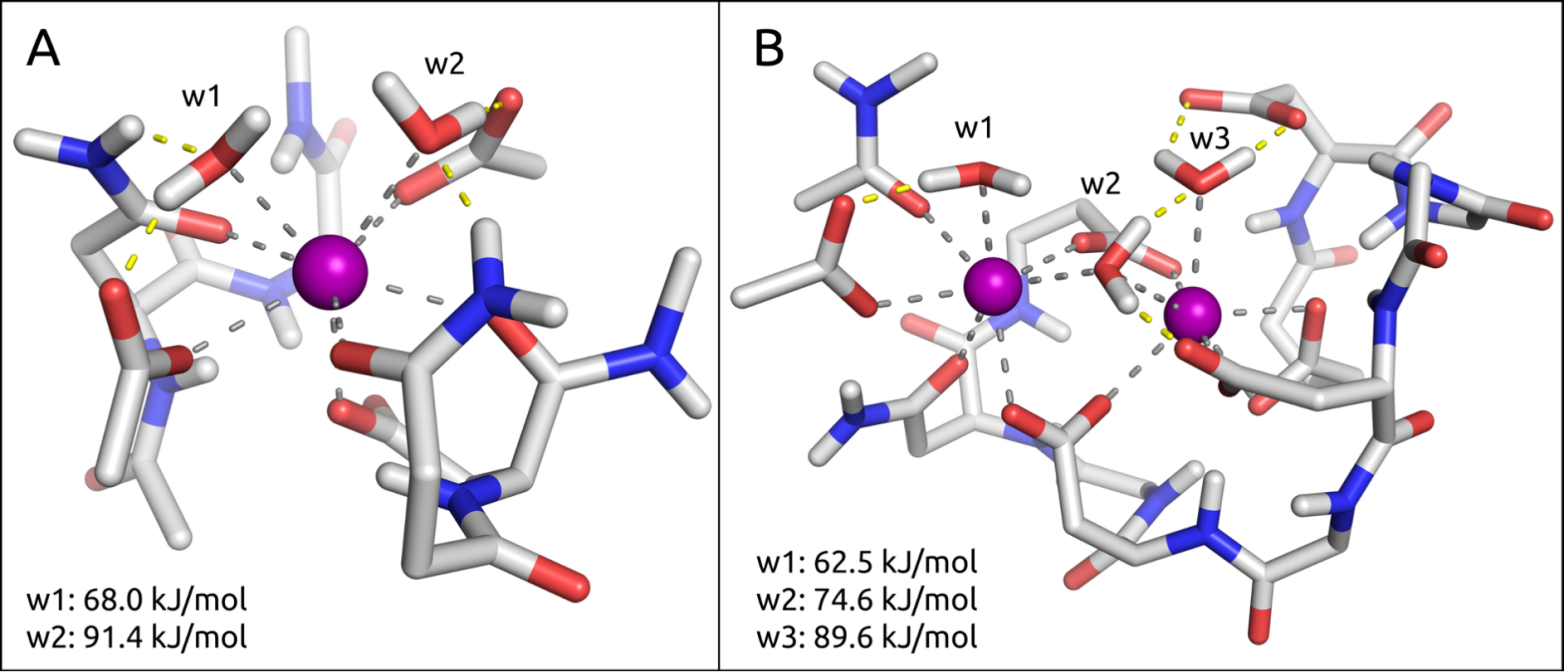
Model view of the binding site interactions of DC-SIGN (**E**) and BC2L-A (**H**) with water. A) The solvent exposed binding site of **E** illustrates two water molecules (w1 and w2) interacting with a single calcium ion, and the cost of desolvation for these waters. B) The buried binding site of **H** exhibits three water molecules (w1–3) bound to two calcium ions, together with their respective desolvation costs.

**The cost of desolvating calcium ions** (Figure 5). Opportunistic bacteria such as *Pseudomonas aeruginosa* or *Burkholderia cenocepacia* have incorporated a second calcium ion into their binding site, coordinating three water molecules which are released into bulk water upon mannose binding and thereby contribute to a favorable entropic effect. The cost to remove one water molecule from a calcium–malonate model system was calculated quantum mechanically (QM) to be 56.9 kJ/mol by Charifson et al. [64]. This is in agreement with electrospray mass spectrometry experiments from Blades et al*.,* who reported water–calcium interaction energies in the range of 62.8 kJ/mol [65]. In-house QM calculations, based on binding site models of DC-SIGN (**E**) and BC2L-A (**H**) (Figure 5), suggest that the average desolvation cost of a single water molecule coordinated to the calcium ion (calculated as a simple difference of the electronic energies of three molecular species: *E*_desolv_ = *E*_receptor···water_ − *E*_receptor_ − *E*_water_) is approximately 77 kJ/mol [66]. Interestingly, the calculated desolvation penalty per calcium ion is more favorable for the binding site of **H** (113 kJ/mol per Ca^2+^), as compared to the one for **E** (159 kJ/mol per Ca^2+^). Similar to the observations made for vicinal hydroxy groups, the rather high desolvation penalty of two calcium ions in the cases of LecB (**G**) and BC2L-A (**H**) (Figure 5B), is in fact reduced when compared to the sum of desolvating two individual calcium ions, again a result of them sharing a common water molecule.

However, the absolute values of the calculated desolvation energies strongly depend on the local environment of each water molecule. For example, w3 in the binding site of BC2L-A (**H**) exhibits a desolvation energy of 89.6 kJ/mol due to the additional interactions to a glutamate and w2. On the other hand, w1 in the exact same binding site is the least costly among the three waters, as it forms fewer interactions and its loss can also be partially compensated by w2 (Figure 5B).

**Figure 6:**
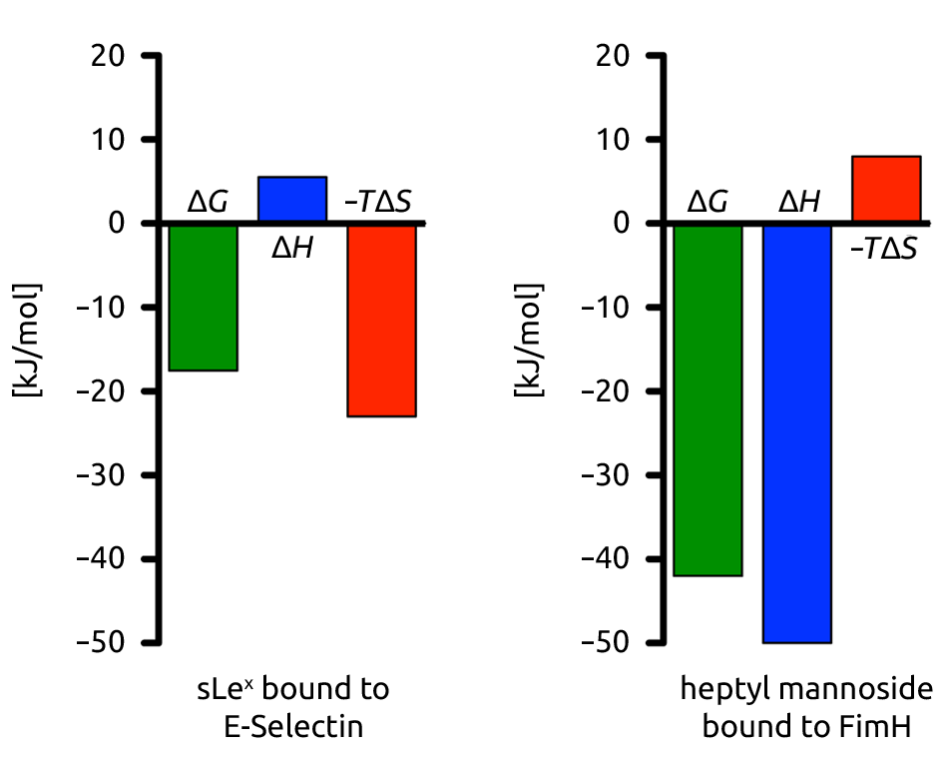
Thermodynamic fingerprints of sLe^x^ bound to E-selectin and *n*-heptyl α-D-mannoside bound to FimH_LD_ (**I**). The sLe^x^–E-selectin interaction is entropically driven, whereas the *n*-heptyl α-D-mannoside–FimH_LD_ (**I**) is enthalpically driven.

**Profiling the pharmacodynamic difference in binding sites**. A comparison of the thermodynamic fingerprints of sLe^x^ interacting with the solvent exposed CRD of E-selectin versus *n*-heptyl α-D-mannoside bound to the buried binding pocket of FimH_LD_ (**I**) represent two different binding scenarios (Figure 2A and B). With the entropically driven sLe^x^ interaction, surface waters are displaced to the bulk and penalized by a positive enthalpy term resulting from a desolvation penalty that is not compensated by the newly formed electrostatic interactions (Figure 6) [67]. According to Dunitz [68], the entropy that can be gained by such waters ranges from 0 kJ/mol for highly mobile waters to 8 kJ/mol for ordered and firmly bound waters. In contrast, the thermodynamic fingerprint of FimH ligands is enthalpically driven because an optimized, stable H-bond network is formed, and as a result, overcompensates the desolvation penalty [69–70].

**Pre-organization vs flexibility**. Carbohydrate–lectin interactions benefit from the low conformational flexibility of pyranoses. This could be impressively demonstrated in a case study comparing a septanose with a *manno*-configured pyranose derivative [71]. Although in both cases an identical H-bond network with the conformationally rigid FimH_LD_ (**I**) was established, the higher flexibility of the seven-membered ring septanose led to a 10-fold loss in affinity. In fact, the number of possible solution conformations was considerably higher for the septanose ligand as compared to the six-membered ring counterpart, effectively increasing the entropic cost of binding to FimH_LD_ (**I**), while the enthalpic fingerprint observed for both ligands was identical.

**Figure 7:**
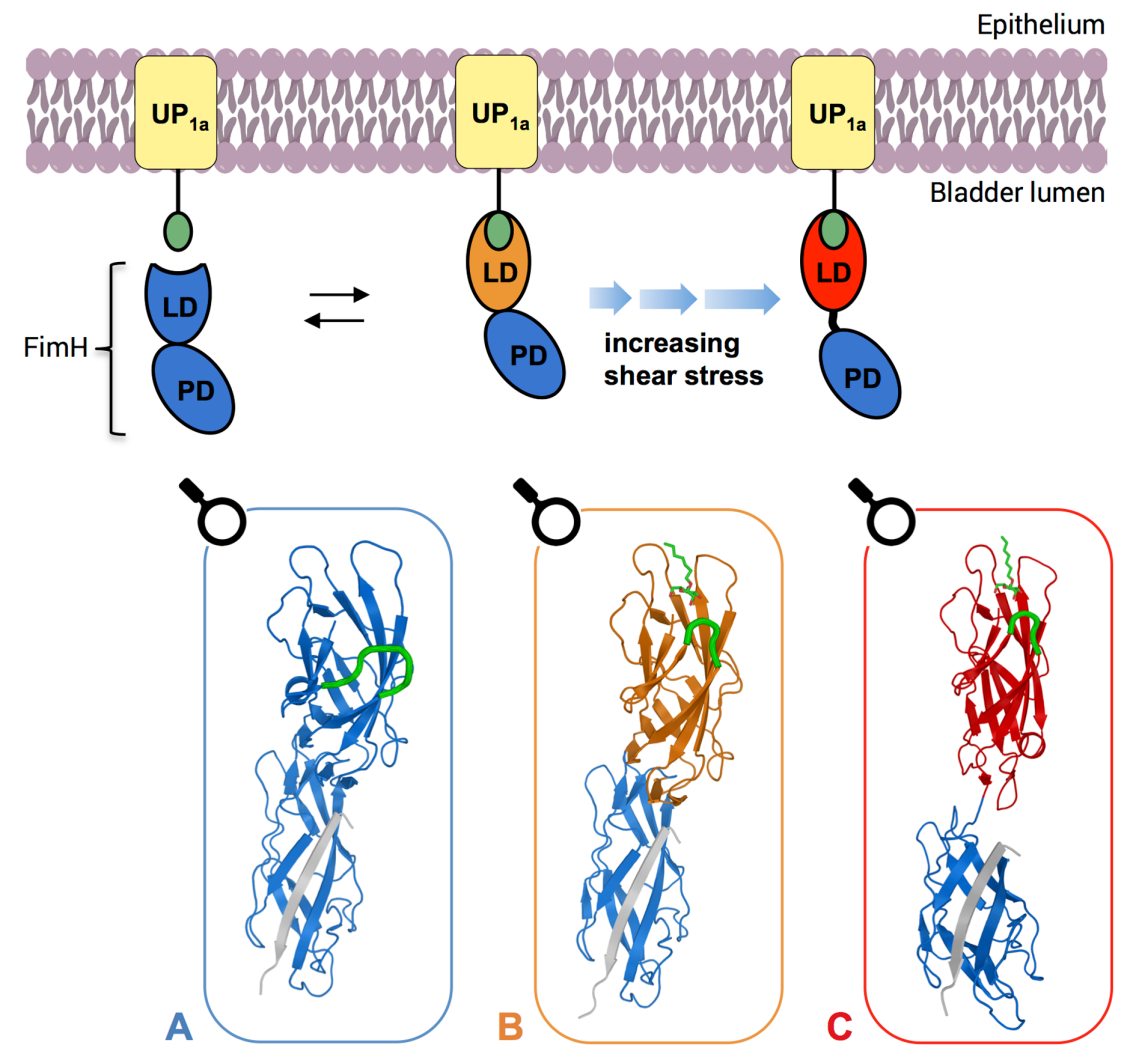
Schematic overview of the conformational changes of FimH (**I**). FimH crystal structures, which correspond to each individual state are shown in boxes. In the absence of urine flow FimH is in the low-affinity conformation, characterized by an open binding pocket and intertwined domains (A, PDB: 4XOD). Upon ligand binding, FimH adopts the medium-affinity conformation (B, PDB: 4XOE), in which a loop (highlighted in green in the crystal structures) closes in the ligand, forming a deep and well-defined binding pocket. As urine begins to flow, the two domains are pulled apart, inducing the high-affinity conformation (C, PDB: 4XOB). LD, lectin domain; PD, pilin domain [72].

However, depending on its needs, UPEC can vary the conformational state of FimH. In the unbound state, FimH exhibits the low-affinity conformation (Figure 7A), which upon binding to mannose, switches to the medium-affinity conformation (Figure 7B). In this state, weak interactions are beneficial because the bacterium can still easily dissociate (slip-bond behavior) and explore its surroundings for optimal nutrient supply. During voiding of the bladder, shear force acts on the FimH protein and pulls the lectin domain (FimH_LD_) away from the pilin domain (FimH_PL_), inducing the high-affinity conformation (Figure 7C), which exhibits an approximate 100-fold higher affinity. Generally, this type of shear force-dependent adhesive bond is known as a catch-bond and in the case of UPECs enables them to evade clearance during micturition. When shear force ceases, FimH reverts back to the equilibrium between low-affinity and medium-affinity conformations [72].

**Figure 8:**
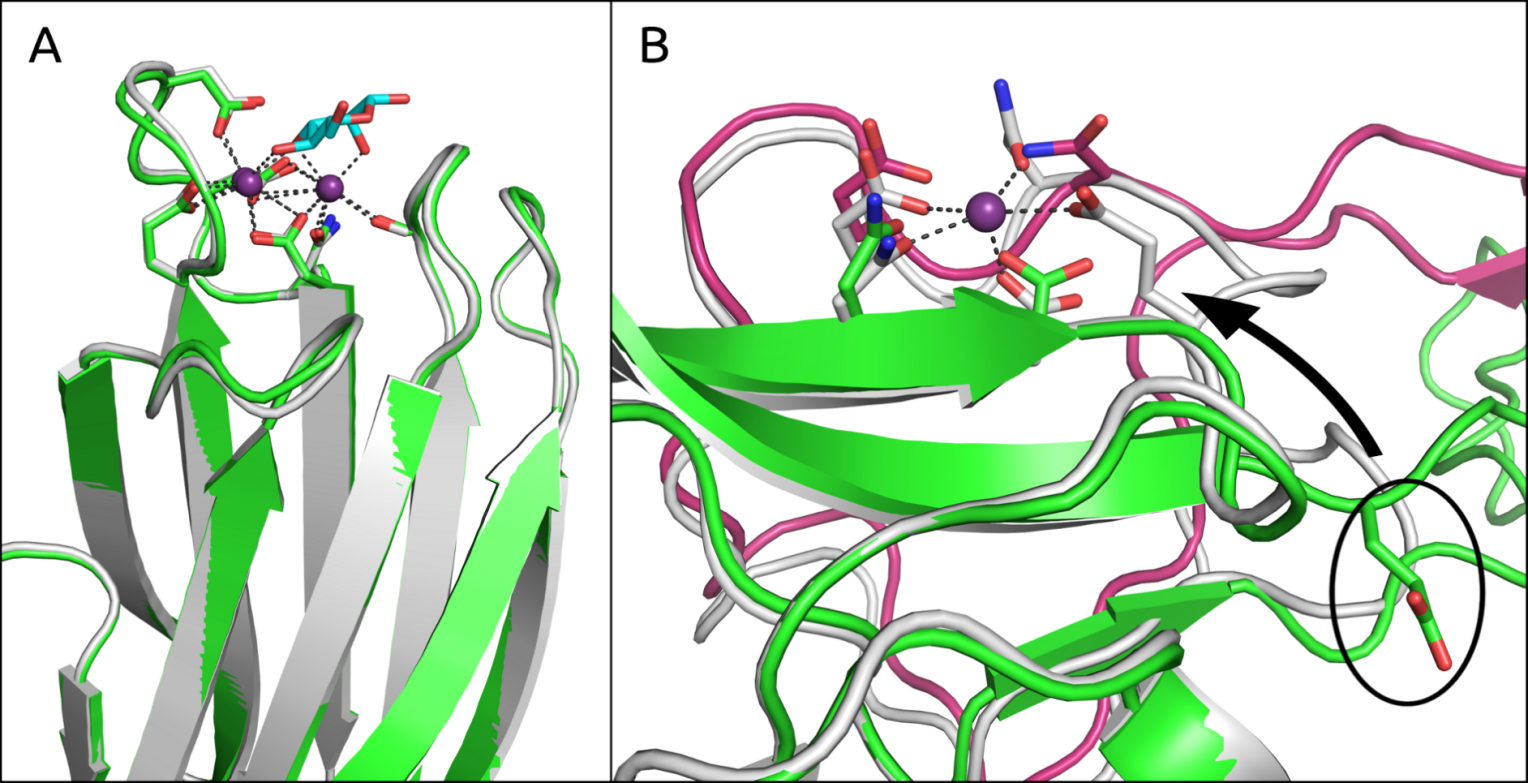
Comparison of the holo (white) and apo (green, magenta) binding sites of LecB (**G**) and BDCA-2 (**A**), respectively. A) The superimposition of both binding sites of **G** reveals nearly identical structures (RMSD of 0.3 Å). B) The binding site of **A** exhibits a flexible loop which enables homodimerization (chain A in green and chain B in magenta), in which a glutamate residue that is essential for Ca^2+^ binding ends up projecting away from the binding site (illustrated for chain A). The situation is mirrored for chain B. Interestingly the loop from chain B closely mimics the binding site of the holo structure (white).

In general, flexible receptors are associated with higher entropic costs resulting from induced-fit binding, which also correlates to facilitated ligand dissociation: due to increased water exposure, the residence time of flexible ligand–lectin complexes is shortened [51]. A comparison of the apo crystal structures of BDCA-2 (**A**) and LecB (**G**) (PDB codes: 3WBP [73] and 1OUX, respectively) to their holo forms excellently demonstrates the entropic costs generated by receptor flexibility. Whereas the binding site of the bacterial lectin **G** does not undergo conformational changes upon ligand binding (RMSD: 0.3 Å; Figure 8A), a conformational change involving a binding site loop allows for the formation of a homodimer of **A** (Figure 8B) [40]. It is believed that this dimer enables transport of the lectin from the Golgi apparatus to cell membranes [73]. Due to a dislocated glutamate in the side chain of the homodimer (Figure 8B), the affinity for calcium binding and therefore also carbohydrate binding is extensively reduced. This remarkable form of inactivation is only possible due to loop flexibility. However, it is also the origin of the low affinity (9.4 mM) towards methyl mannoside (**2**) due to entropic costs associated with the formation of the binding site.

**Multivalency.** Dam and Brewer reviewed the role of density and number of glycan epitopes involved in multivalent carbohydrate interactions for legume lectins as well as for lectins of the innate immune system [74]. As an example, HIV-1 establishes multivalent contacts to DC-SIGN (**E**)-decorated dendritic cells in order to bypass host immune attack. Thus, DC-SIGN plays a key role in the dissemination of HIV-1 by capturing of HIV-1 at entry sites of infection and subsequent transport of the virus to CD4^+^ T cells in lymphoid tissues. The weak monovalent binding affinity of DC-SIGN (**E**) is compensated for by a multivalent display of oligomannosides on viral envelop glycoprotein gp120, facilitating stronger adhesion between dendritic cells and HIV-1 [43,75–76]. This multivalent binding interaction results in an enhancement in binding by several orders of magnitude, from a *K*_D_ of 26 μM for monovalent Man_9_GlcNAc_2_, as compared to 1.7 nM for glycosylated gp120 (25 glycosylation sites) [43,77]. In the case of UPEC, each bacterium contains three to five hundred fimbriae to potentiate multivalency, as each FimH_LD_ (**I**) at the fimbrial tip can interact with mammalian UPIa [78].

Multivalent glycosides have also been investigated in the context of a novel therapeutic approach against viral and bacterial infections [79]. However, carbohydrate valency, spacing, and branching all need to be thoughtfully considered with this class of therapeutics [15,80].

## Conclusion

Mannose-recognizing lectins fulfill a myriad of purposes and depending on the particular biological role either high selectivity and/or high affinity can be required.

On the one hand, lectins of the human immune system tend to exhibit lower affinities due to a higher degree of solvent exposure of their CRDs: fewer H-bond interactions can barely compensate for the high desolvation penalties and constrainment of flexible loop motifs which together contribute to a significant energy penalty upon binding. Nonetheless, these qualities enable ligand promiscuity and can facilitate other features such as the inactivation via homodimerization as exemplified in BDCA-2.

In contrast, bacterial lectins are under constant pressure for survival, hence multiple strategies to ‘get it right the first time’ are employed. For example, the desolvation of a binding site containing two calcium ions costs 113 kJ/mol/Ca^2+^ and therefore is less costly per calcium ion than a binding site containing only a single ion (159 kJ/mol/Ca^2+^; Figure 5). However, in the binding site containing two calcium ions, the ions are able to establish four interactions with the carbohydrate ligand, whereas in the latter example the number of interactions is reduced to two. This leads to an overall enthalpic benefit by forming additional interactions at a reduced cost. In addition, the entropy gained by releasing three water molecules into bulk, as compared to only two, should also be taken into account.

The formation of multiple H-bonds in rigid, buried binding sites is an alternative way to gain enthalpy, and thereby increase affinity. UPEC perfects this approach with the calcium-devoid binding site of FimH_LD_ (**I**). A possible explanation for the lack of a calcium ion in the FimH binding site may relate to the slight acidity of urine (pH 5.5–7.0), with a calcium clearance of 20–300 mg/day [81]. Calcium-dependent lectins require a non-acidic environment, such as found in blood, since at lower pH the glutamate and/or aspartate side chains essential for calcium binding can become partially protonated. Instead, FimH_LD_ forms an extensive hydrogen-bond network in a buried, rigid binding site, which lowers the dielectric constant resulting in better shielded, stronger hydrogen bonds, and also reduces the entropic penalty of binding [82]. In addition, the recently described catch-bond behavior of FimH_LD_ is responsible for a 100-fold increased affinity under selective pressure [70,83]. Together with the multivalency of the interaction this results in the high affinity of **2** to FimH_LD_ (**I**).

The examples apparent in nature of effective mannose recognition domains rely on a combination of partially opposed effects. They nevertheless offer interesting perspectives for the development of carbohydrate-based drugs. One such example of a therapeutic application can be found in a recent novel approach to treating anti-myelin-associated glycoprotein (anti-MAG) neuropathy, a rare, disabling autoimmune disorder. The use of a multivalent glycopolymer mimicking the natural HNK-1 epitope proved to be a valid approach to selectively sequester the autoantibodies associated with anti-MAG neuropathy onset. By applying a multivalent strategy, the inhibitory potential of the monomeric carbohydrate epitope (*K*_D_ 124–793 μM from individual patient sera) could be improved by up to a factor of 230,000 in the multivalent display (*K*_D_ 3.6–5.4 nM/epitope) [84].
